# The Impact of Curriculum Design in the Acquisition of Knowledge of Oncology: Comparison Among Four Medical Schools

**DOI:** 10.1007/s13187-017-1219-2

**Published:** 2017-04-03

**Authors:** Dario Cecilio-Fernandes, Wytze S. Aalders, André J. A. Bremers, René A. Tio, Jakob de Vries

**Affiliations:** 1 Center for Education Development and Research in Health Professions (CEDAR), University of Groningen and University Medical Center Groningen, Antonius Deusinglaan 1, FC40, 9713 AV Groningen, The Netherlands; 2 University Medical Center Groningen, University of Groningen, Groningen, The Netherlands; 3 Department of Surgery, Radboud University Nijmegen Medical Centre, Nijmegen, The Netherlands; 4 Center for Education Development and Research in Health Professions (CEDAR) and Department of Cardiology, University of Groningen and University Medical Center Groningen, Groningen, The Netherlands; 5 Department of Surgery, University of Groningen and University Medical Center Groningen, Groningen, The Netherlands

**Keywords:** Progress test, Oncology knowledge, Knowledge acquisition

## Abstract

Over the past 5 years, cancer has replaced coronary heart disease as the leading cause of death in the Netherlands. It is thus paramount that medical doctors acquire a knowledge of cancer, since most of them will face many patients with cancer. Studies, however, have indicated that there is a deficit in knowledge of oncology among medical students, which may be due not only to the content but also to the structure of the curriculum. In this study, we compared students’ knowledge acquisition in four different undergraduate medical programs. Further, we investigated possible factors that might influence students’ knowledge growth as related to oncology. The participants comprised 1440 medical students distributed over four universities in the Netherlands. To measure students’ knowledge of oncology, we used their progress test results from 2007 to 2013. The progress test consists of 200 multiple-choice questions; this test is taken simultaneously four times a year by all students. All questions regarding oncology were selected. We first compared the growth of knowledge of oncology using mixed models. Then, we interviewed the oncology coordinator of each university to arrive at a better insight of each curriculum. Two schools showed similar patterns of knowledge growth, with a slight decrease in the growth rate for one of them in year 6. The third school had a faster initial growth with a faster decrease over time compared to other medical schools. The fourth school showed a steep decrease in knowledge growth during years 5 and 6. The interviews showed that the two higher-scoring schools had a more focused semester on oncology, whereas in the others, oncology was scattered throughout the curriculum. Furthermore, the absence of a pre-internship training program seemed to hinder knowledge growth in one school. Our findings suggest that curricula have an influence on students’ knowledge acquisition. A focused semester on oncology and a pre-internship preparatory training program are likely to have a positive impact on students’ progress in terms of knowledge of oncology.

## Introduction

Cancer has been the leading cause of death in the Netherlands since 2009. In 2014, 105,000 Dutch people were diagnosed, and approximately 44,808 died because of cancer [1]. The prevalence of cancer in the Netherlands shows an annual increase of 1–3% [2]. Most physicians will encounter many oncology cases throughout their careers. Thus, physicians should be well prepared for the complex diagnosis, long-term treatment, and consequences of cancer.

Nowadays, all medical schools in the Netherlands have a partial or full problem-based teaching curriculum. In a problem-based curriculum, students gain experience in approaching (clinical) problems in an integrated way, taking into account prevention, diagnostics, and the competency to use scientific literature in their treatment strategy. This problem-oriented style of teaching could work very well for oncology, due to its often very complex diagnoses and rapidly changing treatment options [3]. However, several authors found a knowledge deficiency in oncology among first- and second-year students in problem-based teaching universities in the USA and Australia [4–8].

A deficit in knowledge could eventually cause harm to a patient. This knowledge deficit may have various causes, one of which is the way oncology is taught and how it is distributed throughout the curriculum. To better understand the influence of curriculum design, we investigated its effects on the growth of students’ knowledge of cancer in different curricula. Additionally, we compared the growth of students’ knowledge of oncology to that of other medical subjects. Subsequently, we investigated factors that might influence students’ knowledge growth by consulting the curriculum coordinators.

## Methods

To answer our research question, we analyzed progress test data from four different schools using a mixed methods model, more specifically an explanatory sequential design. We used the progress test results of all the eligible students from the universities of Leiden, Groningen, Nijmegen, and Maastricht, who started medical school in September 2007. Subsequently, we interviewed the oncology course directors of each school and examined the universities’ syllabi for each curriculum in order to better understand the differences among the curricula and to explain our quantitative findings.

### The Progress Test

The progress test (PT) consists of four quarterly tests of 200 multiple-choice questions that measure students’ knowledge at graduate level. The questions are based on the Dutch National Blueprint for Medical Education. Each consecutive test includes 200 new items. All schools adhering to the Dutch National Blueprint for Medical Education contribute to the provision of test items in all categories, which are then reviewed by a committee. Thus, none of the students benefit disproportionately, because the writers of the items are equally distributed over the individual schools. The progress test is taken by all medical students of the four Dutch medical schools at the same time [9].

### Course Information

All four medical schools were approached to provide us with their syllabi for curricula implemented in 2007–2013. We examined syllabi for all programs for the presence and degree of problem-based teaching using the following characteristics described by Hmelo-Silver (2004) [10]: (a) the use of problems as the starting point for learning, (b) small-group collaboration with flexible guidance of a tutor, and (c) student-initiated learning. Furthermore, we interviewed the course directors in order to estimate the number of contact hours for pathology and oncology, and how they were positioned in the curriculum.

### Data Analysis

As a specific oncology category was not defined within the progress test, all of the 4800 questions used (over 24 test iterations) were reviewed and classified by WA, based upon the presence or absence of oncology content. A random sample of 50 of the questions, with presence or absence of oncology knowledge, were then independently reviewed by RT and JdV. Since there was an agreement on all questions about the presence or absence of oncology knowledge, it was not deemed necessary to further investigate the inter-rater reliability.

Next, we collected all individual test scores and used these to calculate the average of correct answers for each of the 24 test moments on oncology items over time. Because these single-point values were proven to be unreliable in terms of showing knowledge level in previous research [11], we analyzed the data using mixed-effect models. This method calculates the mathematical function that best explains the data [12]. Additionally, growth curves for oncology items were compared to the curve of the remaining items to approach whether knowledge of oncology exceeds overall knowledge or not.

## Results

Data from all 1440 medical students were retrieved. From those, 321 were from Leiden, 313 from Maastricht, 485 from Groningen, and 321 from Nijmegen, all of which had been admitted through the same, centralized, admission procedure. The results show that progress tests have an average of 7.2% questions concerning oncology. At the start of their studies, students, on average, answered 8% of the oncology questions correctly, whereas at the end of the program, they correctly answered 54% of the oncology questions, on average. At the end point, the percentage of correctly answered questions ranged from 51 to 59%.

**Fig. 1:**
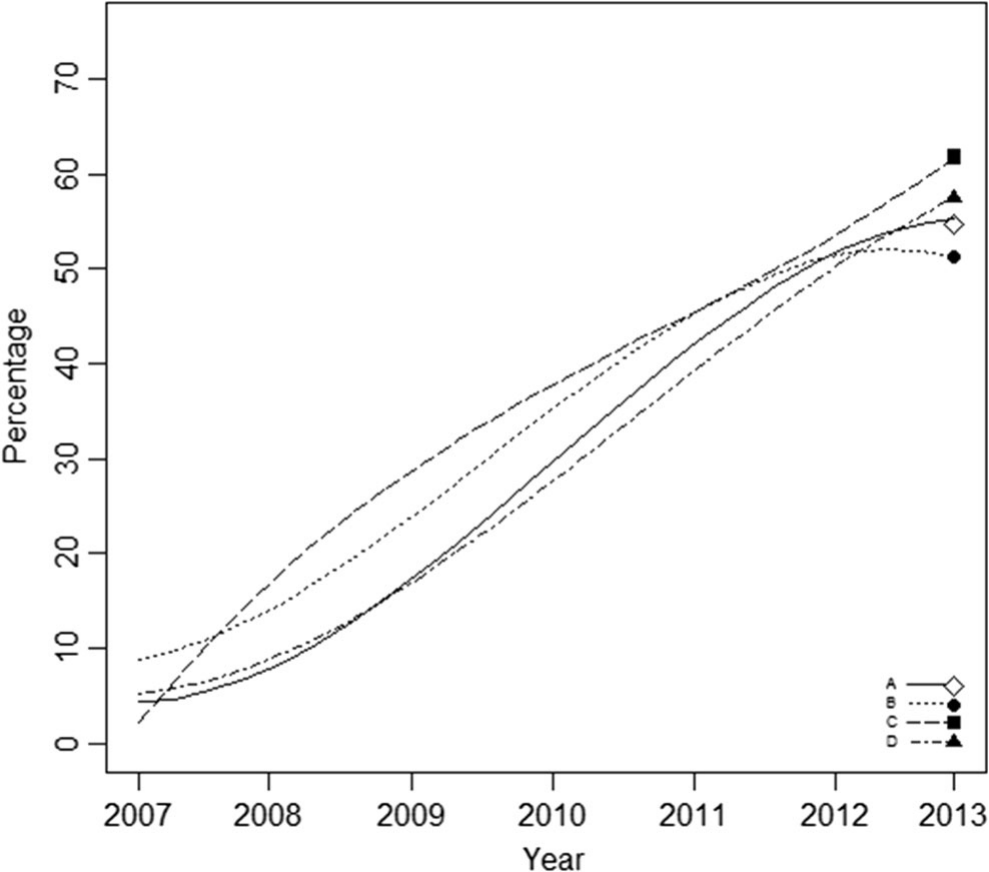
Progression of knowledge of oncology in four different medical schools

When comparing the growth curves of knowledge of oncology among universities (Fig. 1), we found similarities in both linear and quadratic growth models in schools A, B, and D. At the beginning, students had the same knowledge level, and they acquired knowledge at the same pace. In the last year, schools A and B showed a faster decrease in the growth rate of knowledge of oncology compared to schools C and D. Although school B had the fastest initial growth, this rate of growth decreased in the last practical phase of the program, resulting in the lowest end point. School C had a faster initial growth, with a slower decrease in years 3 and 4, and faster growth in years 5 and 6, when compared to the other schools, thus resulting in the highest final level of knowledge of all schools.

Differences found in the four curricula studied, based on their syllabi, are displayed in Table 1. All schools used small tutor groups to support problem-based teaching. Whereas schools B and C showed a teacher-initiated approach, schools A and D showed a problem-based student-initiated approach. In school C, most lectures did not have a clinical problem as a starting point but had the highest number of contact hours in oncology and pathology. Pathology was handled in years 1 and 2 in all cases and was followed by oncology in years 2 and 3. School B had training in oncology solely in year 2. Two schools (C and D) concentrated oncology in one semester, whereas schools A and B combined oncology with other medical subjects in one semester. In three schools, oncology was included in the subjects taught during the fourth year, a pre-internship training program. In school B, we found an absence of pre-internship training-program weeks, meaning that students commenced their internships directly in the fourth year. Schools A and B had the fewest contact hours, followed by school D. School C had more contact hours than the other three medical schools.

**Table 1:** Characteristics of four Dutch medical curricula

	Degree of problem-based teaching	Oncology concentrated in one semester	Pre-internship training in oncology	Estimated contact hours for pathology	Estimated contact hours for oncology
School A	++	−	+	24	48
School B	+	−	−	32	40
School C	+/−	+	+	48	56
School D	++	+	+	32	48

**Fig. 2:**
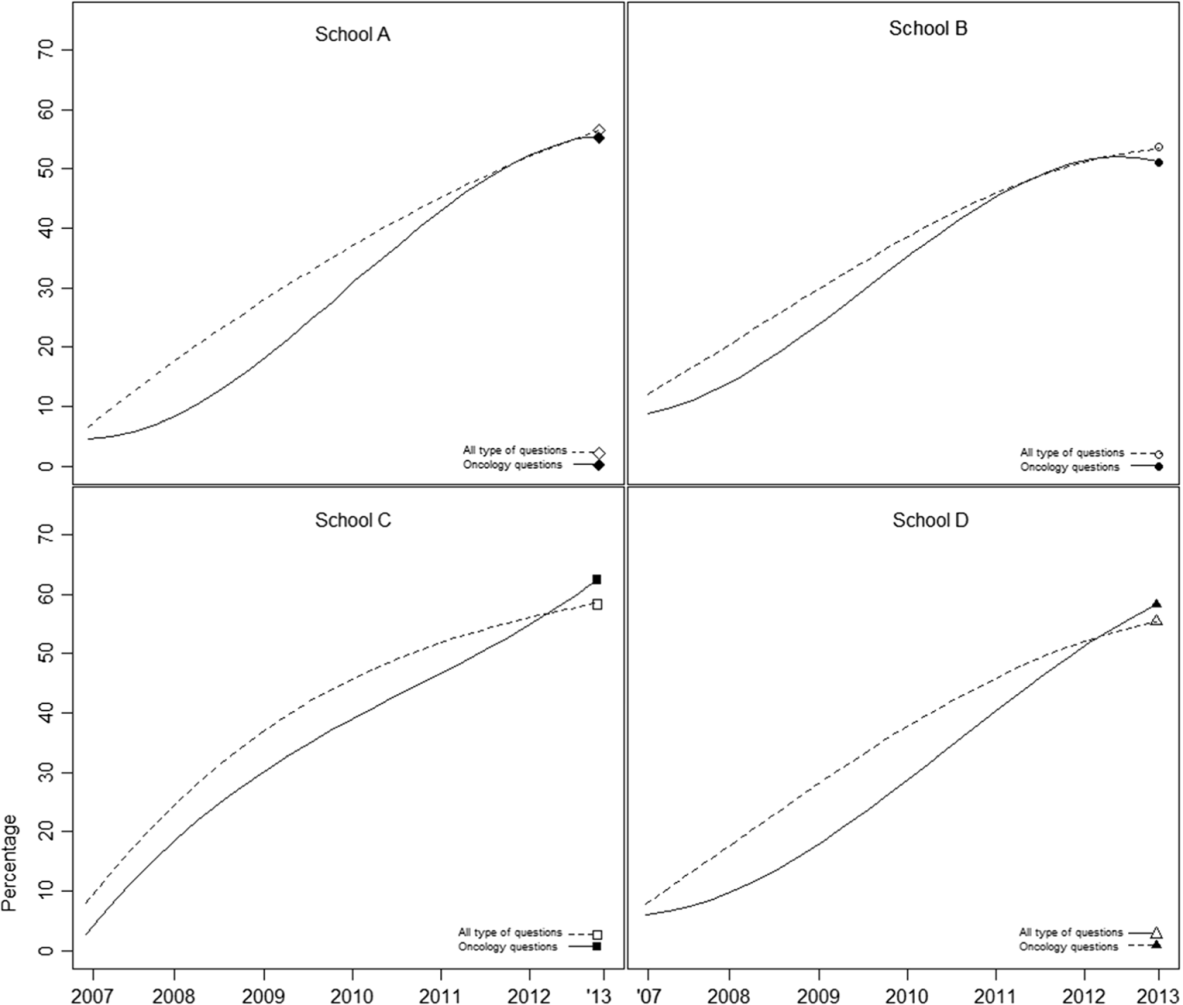
Knowledge of oncology items in four different universities compared to other knowledge

Figure 2 depicts the growth curves of knowledge of oncology compared to overall knowledge, calculated within medical schools. In all groups, there was a significant difference in linear, quadratic, and cubic growth between knowledge of oncology and overall knowledge. At the end points, schools C and D scored higher on oncology, whereas schools A and B scored lower on oncology compared to overall knowledge. However, all schools scored lower on oncology throughout most of the curricula.

## Discussion

In this study, we investigated the influence of four different curricula on students’ acquisition of knowledge of oncology. As expected, the curriculum has an impact on how students acquire their knowledge of oncology. It seems that concentrating the teaching of oncology in one semester is a more important factor in terms of knowledge acquisition than the type of teaching method. Similar results were found in a previous study, in which students who had concentrated oncology block-training performed better than students who had oncology training throughout the curriculum [13].

Two major curriculum design differences might have influenced students’ knowledge growth. First, the type of teaching styles: Schools A and D used a problem-based learning approach, whereas schools B and C use a mix of traditional and problem-based learning approaches. One would imagine that being required to solve cases would facilitate students’ acquisition of a knowledge of oncology. It seems, however, that the type of teaching style plays no significant role, since our results demonstrate no clear difference in terms of teaching styles. Second, schools C and D had more contact hours in pathology and oncology than schools A and B did. A closer look, however, shows that the number of contact hours for teaching oncology in schools A and D were the same, while school A had more contact hours for pathology than school D. This would suggest that the number of contact hours involving basic knowledge (pathology) might lead to a better acquisition of more complex knowledge (oncology). Based on our findings, the number of contact hours may have contributed to students’ growth in knowledge about oncology.

Schools A and B scored lower on oncology questions at the end point compared to schools C and D, and also when compared to overall knowledge. In addition to the absence of concentrated teaching of oncology, schools A and B also taught oncology integrated with other medical subjects. Furthermore, school A had a pre-internship training program, whereas school B did not. This may explain why school B showed a faster decrease in knowledge of oncology. A pre-internship training program would allow students to refresh their knowledge, which would lead to better retention. Alternatively, an explanation could be that some students have less contact with oncology cases during internships than others [14].

Schools C and D scored higher on oncology questions at end point compared to the other two schools and also compared to overall knowledge. Although their teaching styles differed considerably, schools C and D both taught oncology in a concentrated block of 4 weeks, instead of dispersing it among other medical subjects. Whereas students who have oncology spread out may not yet have acquired the knowledge necessary to fully understand more complex cases, students who have a block of oncology teaching may have already acquired the necessary knowledge. This is in line with previous research showing that medical students need to acquire basic factual knowledge before they can apply it [15]. For instance, a concentrated oncology block could focus more on disease prevention and the importance of early diagnostics rather than on treatment. Compared to other medical subjects, students must learn the importance of the staging system and its implications for the prognosis. Focusing on an “oncological way of thinking,” involving basic knowledge of oncology and its application in a concentrated oncology block, might be more effective in teaching oncology.

Our study has a few limitations. The retrospective character of this study does not allow us to control for variables that might have influenced our findings. However, it offers a unique opportunity to look closely at the real-life situation, which is often different from laboratory studies. The use of the Dutch progress test results may be a limitation since it measures students’ knowledge at end level. However, the use of the Dutch progress test, being a summative test, eliminates the bias of students’ willingness to participate, and it is possible to investigate students’ knowledge growth throughout their undergraduate training with a minimum of confounding through variation between tests.

## Conclusions

Based on our findings, we conclude that more contact hours, a focused semester on oncology, and a pre-internship preparatory training program are likely to have a positive impact on students’ progression in knowledge of oncology.
